# RiboNT: A Noise-Tolerant Predictor of Open Reading Frames from Ribosome-Protected Footprints

**DOI:** 10.3390/life11070701

**Published:** 2021-07-16

**Authors:** Bo Song, Mengyun Jiang, Lei Gao

**Affiliations:** 1 Guangdong Provincial Key Laboratory for Plant Epigenetics, College of Life Sciences and Oceanography, Shenzhen University, Shenzhen 518060, China; 2 Shenzhen Branch, Guangdong Laboratory of Lingnan Modern Agriculture, Genome Analysis Laboratory of the Ministry of Agriculture and Rural Affairs, Agricultural Genomics Institute at Shenzhen, Chinese Academy of Agricultural Sciences, Shenzhen 518120, China; jiangmengyun@caas.cn; 3 State Key Laboratory of Crop Stress Adaptation and Improvement, School of Life Sciences, Henan University, Kaifeng 475004, China; 4 Shenzhen Research Institute of Henan University, Shenzhen 518000, China

**Keywords:** Ribo-seq, ribosome profiling, ORFs, small ORFs, periodicity, RPFs

## Abstract

Ribo-seq, also known as ribosome profiling, refers to the sequencing of ribosome-protected mRNA fragments (RPFs). This technique has greatly advanced our understanding of translation and facilitated the identification of novel open reading frames (ORFs) within untranslated regions or non-coding sequences as well as the identification of non-canonical start codons. However, the widespread application of Ribo-seq has been hindered because obtaining periodic RPFs requires a highly optimized protocol, which may be difficult to achieve, particularly in non-model organisms. Furthermore, the periodic RPFs are too short (28 nt) for accurate mapping to polyploid genomes, but longer RPFs are usually produced with a compromise in periodicity. Here we present RiboNT, a noise-tolerant ORF predictor that can utilize RPFs with poor periodicity. It evaluates RPF periodicity and automatically weighs the support from RPFs and codon usage before combining their contributions to identify translated ORFs. The results demonstrate the utility of RiboNT for identifying both long and small ORFs using RPFs with either good or poor periodicity. We implemented the pipeline on a dataset of RPFs with poor periodicity derived from membrane-bound polysomes of *Arabidopsis thaliana* seedlings and identified several small ORFs (sORFs) evolutionarily conserved in diverse plant species. RiboNT should greatly broaden the application of Ribo-seq by minimizing the requirement of RPF quality and allowing the use of longer RPFs, which is critical for organisms with complex genomes because these RPFs can be more accurately mapped to the position from which they were derived.

## 1. Introduction

Small open reading frames (sORFs) encode small peptides shorter than 100 amino acids [1] and are usually neglected in the annotation of genomic coding sequences (CDSs) due to their short lengths. The translation of sORFs can be initiated by AUG or near-cognate codons (CUG, GUG, UUG) [2,3]. However, in most, if not all, of the released reference genome sequences, only AUG is recognized as the start codon for CDS initiation, leading to poor sORF annotation. The functions of sORFs remain poorly understood, and sORF annotation based solely on DNA sequences is difficult because numerous nonsense sORFs can arise randomly by chance. Despite the accuracy of the experimental approaches for sORF identification, such as gene knock-out and fusion with visible or detectable tags, the labor and time costs are high [1,4]. Nevertheless, the growing number of genome and transcriptome datasets in various organisms have allowed the identification of several sORFs based on sequence similarity based on the assumption that functional sORFs are evolutionarily conserved [5].

Ribo-seq, which profiles the mRNA footprints protected by ribosomes, has facilitated the genome-wide annotation of translated ORFs including sORFs. For this technique, ribosome-bound mRNA is digested by RNase, and the resulting monosomes are isolated before the sequencing of the ribosome-protected mRNA fragments (RPFs), followed by the allocation of translated codons on mRNA by calculating the distance between the 5′ end of the RPFs and peptidyl-sites (P-sites) [6]. The offset of RPFs of different sizes can be trained from the reads that map to regions containing start codons (P-sites) or stop codons (aminoacyl-sites, A-sites), where ribosomes always stall.

Pipelines utilizing different algorithms have been developed to predict unannotated ORFs in various organisms (reviewed in [7]). These methods assume that most RPFs are uniform in size and offset, therefore relying on the periodicity and resolution of RPFs. Typically, a ribosome occupies 28 nt on mRNA with an offset of 12 nt from the 5′ terminus to the P-site being translated [8,9]. This prerequisite may be satisfied in model organisms [2,3,9] but may not be easy to achieve in many other non-model organisms. Furthermore, even in model organisms, isolating membrane-bound ribosomes and extracting high-resolution RPFs are still major challenges. In a study in *Arabidopsis*, Li et al. [10] extracted membrane-bound RPFs, and the sizes varied widely from 15 to 35 nt, with a peak at 32 nt, and poor periodicity. Analysis of such noisy data will lead to inaccuracy or errors if the methods used for prediction are dependent on the periodicity of the RPFs. The technical challenge of extracting high-resolution RPFs has greatly limited the applications of Ribo-seq, and many researchers are working to improve protocols for monosome preparation and isolation [9]. At the same time, a noise-tolerant method is also needed to expand the applicability of Ribo-seq to a broader range of organisms.

Here we describe RiboNT, a method tolerant to noise arising from RPF size, offset and periodicity. Besides the support from RPFs, the probability of each triplet as a codon within a candidate ORF is also considered in RiboNT. This pipeline automatically balances the weight of these two lines of evidence/support and comprehensively evaluates the probability of an ORF for a given sequence. We implemented this method on a noisy dataset derived from *Arabidopsis* membrane-bound polysomes and found 13 ncsORFs, many of which are evolutionarily conserved in diverse plant species.

## 2. Materials and Methods

### 2.1. Design of RiboNT

RiboNT predicts ORFs primarily based on features describing ribosome behaviors and CDS characteristics. The former is also usually implemented in other RPF-based ORF finders [7], with the latter used for genomic ORF annotation. Computational prediction of ORFs from DNA sequences alone is insensitive to sORFs as the signal would arise by chance. In contrast, the periodic occupancy of ribosomes on mRNA provides strong evidence of ORF translation, and the footprints can be used to accurately predict the translated ORFs, including sORFs. However, the RPF-dependent approach relies heavily on good RPF periodicity and resolution. In this study, we attempted to integrate these two strategies to balance the weight of ribosome occupancy and sequence characteristics. For a given candidate ORF, we evaluated whether the distribution of RPFs on frame 0 was larger than that on frame 1 and 2, and whether the triplets in frame 0 were more probable as codons than those in frame 1 and 2. In total, we performed four Student’s *t*-tests (RPF: frame 0 vs. 1, frame 0 vs. 2; codon usage: frame 0 vs. 1, frame 0 vs. 2) and combined the *p* values with varied weights, which were automatically determined by the quality of the RPFs. A final *p* value was generated for each candidate ORF, and those with *p* values less than 0.001 were identified as bona fide translated ORFs. 

**Figure 1:**
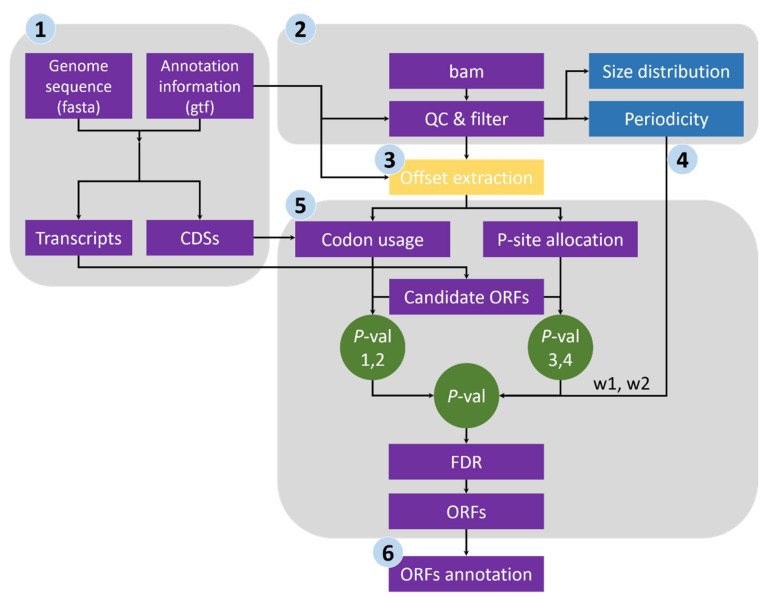
The RiboNT workflow consists six steps. Step 1: assemble transcripts according to the genome annotation information and extract candidate ORFs; step 2: examine the quality of RPFs and filter out low-quality RPFs; step 3: calculate the offsets to the start codon for RPFs in each size; step 4: balance the weight between RPFs and codon usages according to the periodicity of RPFs; step 5: identify translated ORFs from candidate ORFs by combing four student’s *t*-tests (RPF depth, frame 0 vs. 1, 2; codon usage, frame 0 vs. 1, 2); step 6: classify the predicted ORFs into different classes.

RiboNT takes in three input files: the reference genome sequence (in fasta format), the genome annotation (in gtf format) and reads alignment (in bam format), and process them in six steps as shown in Figure 1.

**Figure 2:**
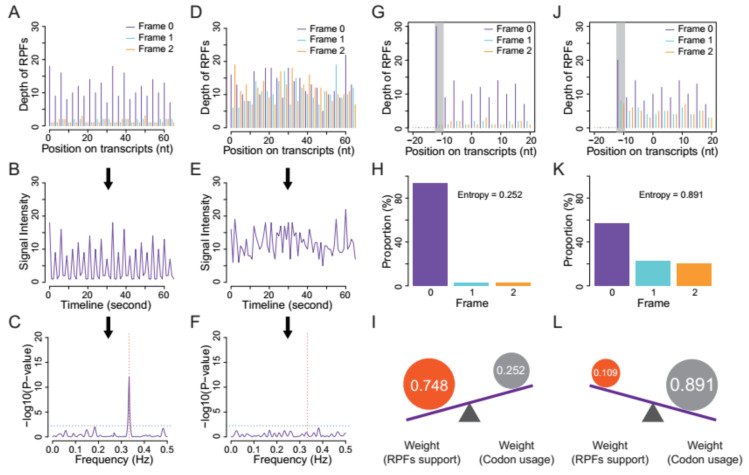
The strategy of RPFs filtering and weighing. (**A**) The distribution of high-quality RPFs on CDSs. (**B**) The depth distribution of RPFs shown in (**A**) is transformed into a wave function by connecting the vertices, with the coordinates on the CDSs converted into a timeline in second. (**C**) The resulting wave was subjected to a F-test implemented in “multitaper”, an R package. RPFs showing significance (*p* <= 0.01) at frequency of 0.33 Hz (the period recurs every 3 s) were retained. RPFs without periodicity (**D**–**F**) do not satisfy these criteria. The horizontal (gray) and vertical (red) dotted lines in (**C**) and (**F**) indicate the position of *p* = 0.01 and Frequency = 0.33 Hz, respectively. (**G**) The first RPFs shown in CDSs represent the mRNA fragments protected by ribosomes translating the start codon. Their offsets to the start codon were determined by the distance from its 5′ terminus to the translated P-sites. (**H**) RPFs predominantly located on one of these frames would result in lower overall entropy and will be weighted more (**I**) in the identification of ORFs. On the other hand, (**J**) RPFs with weak periodicities have higher overall entropy (**K**) and will be weighted less (**L**).

**Figure 3:**
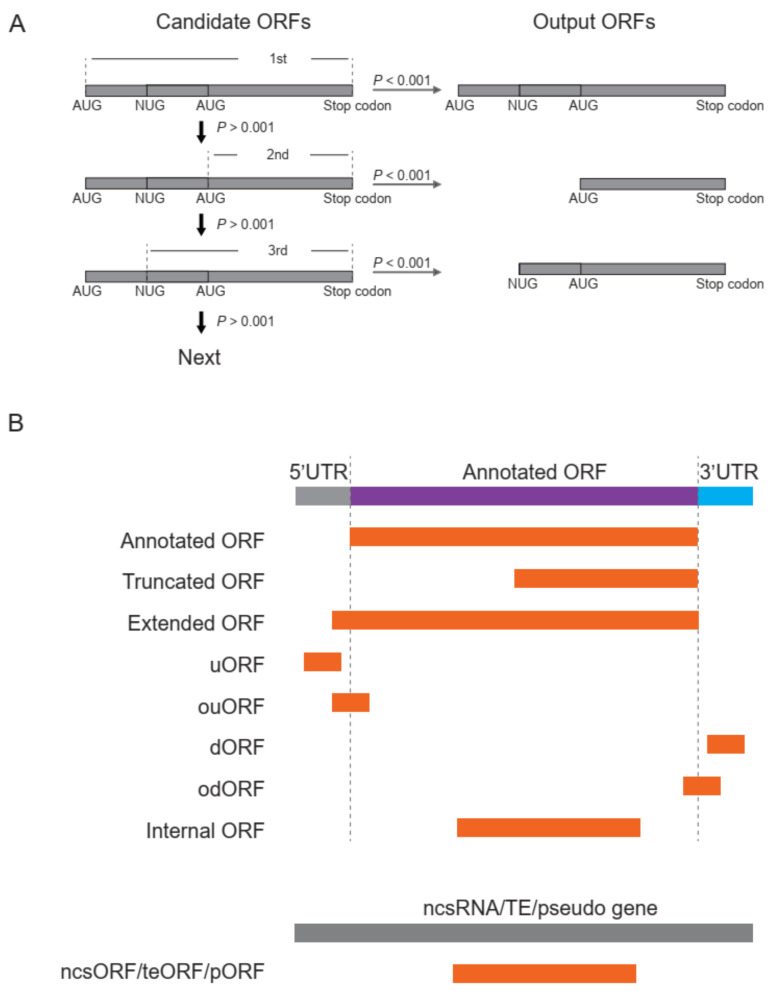
Searching and classification of ORFs. (**A**) Stepwise search for the longest ORF candidates with a priority to those start with AUG. (**B**) Classification of identified ORFs according to their positions relative to the annotated ORFs.

1. Sequence extraction of annotated CDSs and ORF candidates. We first extracted the CDS sequences from the reference genome file according to the annotation data and subsequently calculated the genome-wide usage of codons in the annotated CDSs. Transcript sequences were also extracted, and all potential ORFs (beginning with start codon AUG or NUG, ending with a stop codon and having a multiple-of-three length) were retained (Figure 1).
2. Quality evaluation of RPFs. RPFs mapped to the first 60 bp of a CDS were used to evaluate RPF periodicity (Figure 2A). RPF filtering was performed using an F-test implemented in the ‘multitaper’ R package [11], which was also used for ORF prediction in RiboTaper [12]. Briefly, we first converted the RPF position and depth along the CDS into a time axis (in seconds) and signal intensities, respectively (Figure 2B). The ‘multitaper’ R package (version 1.0-14) [11] was applied to extract the spectrum and frequency of this ‘signal’. A frequency of 0.33 Hz indicates that the peak of the ‘signal’ appears every three seconds (nucleotides in CDS). An F-test implemented in ‘multitaper’ was performed to calculate the *p* values for all the frequencies extracted from this ‘signal’. In this pipeline, RPFs with a *p* value less than 0.01 at a frequency of 0.33 were selected as periodic RPFs for downstream steps (Figure 2C); those that did not satisfy these criteria (Figure 2D–F) were discarded.
3. Offset extraction of RPFs. The offsets to the P-site were counted for RPFs in each size class using the RPFs that overlapped with the start (P-site) or stop codon (A-site). Translation initiates from the start codon, so the largest distance from the RPF 5′ terminus to the start codon is the offset to the P-site (Figure 2G–L). As noisy RPFs may show different offsets, instead of a unique offset for each size (Figure 2J), three offsets with corresponding probabilities were calculated for each RPF size using the RPF depths at the first three positions (Figure 2H,K).
4. Weight balance. We integrated the support from RPFs and codon usage in this pipeline. One underlying principle is that RPFs with greater periodicity are assigned greater weight; if the periodicity is poor, greater weight should be given to the support of codon usage. We used the differences in RPF distribution on frame 0, 1 and 2 to measure the degree of periodicity. RPFs with high periodicity were preferentially distributed on one of these frames with very high proportions. The diversity was calculated using the following formula for entropy:
  (1) $$\mathrm{Entropy}\;=\sum_{i=0}^{2}-\;p_{i}\;\log_{n}\;\left(p_{i}\right)$$
  where *i* denotes the frame (0, 1 and 2), *p_i_* denotes the proportion of RPFs distributed on frame *i*, and *n* is logarithmic base. To constrain the interval of entropy between 0 and 1, we set *n* = 3 for the pipeline. From this formula, RPFs with greater periodicity will have lower entropy (Figure 2H,K). With an even distribution of RPFs among the three frames (the lowest periodicity), *p_i_* constantly equals 1/3 when *i* = 0, 1 and 2, resulting in an entropy of 1. By contrast, exclusive accumulation of RPFs on only one of the frames (the greatest periodicity), e.g., frame 0, would result in an entropy of 0. Finally, we used the entropy value to weigh the contributions from codon usage and [1 − Entropy] to weight those from RPF support (Figure 2I,L).
5. ORF identification. RPFs were proportionally allocated to their corresponding P-sites according to the offsets extracted in step 3. The RPF depth was transferred to P-site depth, and the values were normalized to a Z-score before two Student’s *t*-tests were performed to determine whether the depths at frame 0 were significantly greater than those at frame 1 and 2 for a given ORF candidate. Similarly, the codon usage was also assigned to each triplet in the sequence of a given ORF candidate, and two additional Student’s *t*-tests were performed to determine whether the triplets at frame 0 had greater usage than those at frame 1 and 2. The four *p* values were weighted according to the RPF periodicity calculated in step 4 and combined using a weighted chi-square method [13] with the following formula:
  (2) $$M=-2\times \sum_{i=1}^{4}\;-\;w_{i}\;In\;\left(p_{i}\right)$$
  where *p_i_* denotes the *p* values and *w_i_* denotes the weight for *p_i_* and satisfies ∑wi = 1. The *M* value is distributed as 2*χ*^2^*_k_*/*k*, where *k* indicates degrees of freedom, determined according to the weights and correlation between the four tests. According to the algorithm described in [13], *k* ranges from 2 to 8 in this study. When the four *p* values are the same, *k* = 2, suggesting these four tests are identical; when the four tests are independent, *k* = 8, which is equivalent to that used in Fisher’s method (*k* = 2n, where n is the number of *p* values to be combined). We rejected *H_0_*, a non-translated candidate ORF, if *M* > 2 × *χ*^2^*_k_*_, 1-α_/*k*. The value of *α* was set to 0.001 in this pipeline, candidates with *p* values less than 0.001 were retained, and the false discovery rate (FDR) was set to 0.0001. For each stop codon, stepwise searching for the longest candidate is performed in RiboNT, which will stop and output the candidate when its *p* value is smaller than *α*. The start codon(s) (AUG by default) can be optionally customized; however, considering the higher usage of AUG [2], RiboNT would still output ORFs starting with AUG with higher priority even if other codons were selected (Figure 3A).
6. Classification of predicted ORFs. To ensure consistency with the categories reported in previous works [12,14], several criteria from those studies were incorporated into RiboNT, which classifies the predicted ORFs into 11 categories: (i) annotated ORF, ORFs identical to annotated ORFs; (ii) truncated ORF, ORFs with the same start or stop codon but shorter than the annotated sequence; (iii) extended ORF, ORFs with the same start or stop codon but longer than the annotated sequence; (iv) uORF, upstream ORF, ORFs located in 5′-UTRs; (v) ouORF, overlapped uORF, ORFs located in 5′-UTRs and overlapping an annotated start codon; (vi) dORF, downstream ORF, ORFs located in 3′-UTRs; (vii) odORF, overlapped dORF, ORFs located in 3′UTRs and overlapping an annotated stop codon; (viii) ncsORF, ORFs located in non-coding RNAs, with ORFs predicted from genes without any annotated CDSs also classified as ncsORFs; (ix) internal ORF, ORFs located inside annotated ORFs; (x) teORF, ORFs located in transposable elements; and (xi) pORF, ORFs on pseudogenes (Figure 3B).

### 2.2. Comparison between RiboNT and Other Predictors

Different predictors have been developed for different purposes (reviewed in [7,15]). In this study, we compared RiboNT with RiboTaper (v1.3) [12], RiboCode (v1.2.11) [14] and RiboWave (v1.0) [16] for the annotation of CDSs and translation initiation sites. RiboWave reported errors during the annotation file reation stages for yeast and therefore was not tested on the yeast datasets. The predicted ORFs identical to those annotated in the genome were counted as true positives, and the others were considered false positives. In each comparison, the precision (Number of true positives/Total number of predicted ORFs), recall (Number of true positives/Total number of annotated ORFs) and F-score [2 × Recall × Precision/(Recall + Precision)] were calculated, and F-scores were used to comprehensively assess the performance of each predictor.

Two datasets derived from human and yeast (*Saccharomyces cerevisae*) were downloaded from NCBI (accessions SRR1630833 [2] and SRR5681104 [3]). The reads from these datasets were processed by trimming the adaptors and retaining only the trimmed reads. To compare the performance of the above predictors on noisy datasets, we artificially introduced different amounts of noise to the resulting RPFs by randomly trimming 1 to 5 nucleotides from the beginning or end of the RPFs. These noisy RPFs combined with the original datasets, were aligned to the human (Ensembl release 70) and yeast (*S. cerevisae*, S288C) reference genomes using STAR (v. 2.5.3a) [17] with default parameters and were used for the comparisons. These noisy RPFs were assigned with weights ranging from 0.64 to 0.18 for the prediction of human ORFs and from 0.61 to 0.31 for the prediction of yeast ORFs, and the weights of codon usage increased correspondingly (Table S1).

### 2.3. Validation of Predicted ORFs Using MS Datasets

Protein mass spectrometry (MS) datasets of human HEK293 cells, *S. cerevisiae* and *A. thaliana* were downloaded from the PRIDE archive (accessions PXD003133 [18] for human HEK293, PXD010868 [19] for *S. cerevisiae* and PXD009484 [20] and PXD009274 [21] for *A. thaliana* seedlings). The files of raw data derived from the wild-types of these species were obtained, and MaxQuant [22,23] with default parameters was used to search the peptides encoded by the ORFs identified by the different pipelines using RPFs with different amounts of noise.

### 2.4. Identification and Analysis of ORFs from Human and A. thaliana Low-Quality RPFs

RiboNT, RiboCode, RiboTaper and RiboWave were used to predict ORFs from a noisy dataset of human RPFs [24]. This dataset was generated using a simplified and inexpensive method involving the digestion of crude cellular extracts with micrococcal nuclease. The resulting RPFs were longer but had poor periodicity. The RPFs were aligned to the reference genome and used for ORF prediction as described above.

RiboNT, RiboCode and RiboTaper were used to predict ORFs from a low-quality RPF dataset derived from *A. thaliana* membrane-bound polysomes [10]. The identified ORFs were validated using MS datasets of proteins extracted from wild-type *A. thaliana* seedlings as described above. The peptides encoded by the ncsORFs identified from this dataset were used to search for their homologs in various plant genomes, obtained from Phytozome (https://phytozome.jgi.doe.gov, accessed on 10 June 2018), using the methods and criteria described in [9]. Briefly, genome assemblies of 16 species in different lineages from ferns to mono- and eudicots species, including five other cruciferous species, were downloaded from Phytozome. The homologs in these genomes were identified by sequence alignment using tBLASTn with default parameters and E-value threshold set to 0.1. The sequences hit to the queried peptide (ncsORFs) with more than 30% coverage were retained and the sequence identities were recorded, according to which the ncsORFs were clustered and visualized using the ‘pheatmap’ R package.

## 3. Results

### 3.1. Identification of Annotated ORFs

**Figure 4:**
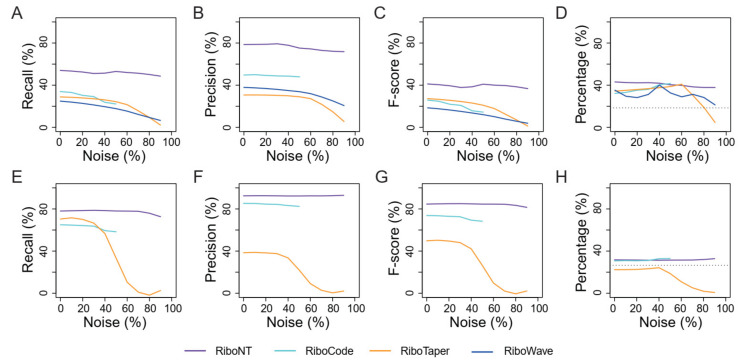
Performance of RiboNT, RiboCode and RiboTaper in detecting annotated ORFs. The recall (**A**), precision (**B**), F-score (**C**) and percent support from MS datasets (**D**) of annotated ORFs identified by RiboNT (purple), RiboCode (cyan) and RiboTaper (orange) in human (**A**–**D**) and yeast (**E**–**H**) datasets using RPFs with varying amount of noise (0–90%). The horizontal dotted lines in (**D**) and (**H**) indicate the percentage of MS-validated ORFs in the reference genome.

Gene CDSs and ORFs are well annotated in many genomes. Particularly in model organisms, many ORFs have been experimentally validated by gene knock-out, over-expression or fusion with visible or detectable tags such as green florescent protein (GFP). We reasoned that a reliable ORF predictor should be able to recover most of the annotated ORFs in the transcriptome. We first compared the performance of several tools for the prediction of annotated human and yeast ORFs using high-quality RPFs. RiboNT predicted 48,979 and 5655 ORFs, of which 78.93% (38,659) and 92.43% (5227) were identical to the annotated ORFs in the human and yeast genomes, respectively. Furthermore, 40.44% (38,659 of 95,587) and 78.05% (5227 of 6697) of all the annotated ORFs in the human and yeast genomes were successfully recovered by RiboNT. RiboCode also recovered most of the annotated ORFs in yeast and human with adequate precision (Figure 4A–C,E–G; Tables S2 and S3). RiboTaper and RiboWave predicted fewer ORFs; the former most likely reflects its requirement of at least 50% of RPF-supported P-sites [12].

We further assessed the recall and precision of these tools when 10% to 90% noise was artificially introduced into the datasets. With increasing noise, both recall and precision decreased in RiboTaper, particularly when the level of noise exceeded 40% (Figure 4A–C,E–G). RiboCode stopped reporting when the noise was greater than 50%; below this level, the recall of RiboCode decreased slightly as noise increased, and its precision was hardly affected. The F-score of RiboWave decreased as a function of the noise (Figure 4A). For RiboNT, neither recall nor precision was affected by noise lower than 70% (Figure 4C,G). When noise exceeded 70%, RiboNT had minor decreases in recall and F-score for the prediction of yeast ORFs.

The peptides encoded by the ORFs identified in each of these tests were pooled and searched using the MS protein data of human (HEK293 cells) or *S. cerevisiae*. The results suggest that without noise, higher percentages of peptides were supported by MS data in the dataset of predicted ORFs, compared to that of the reference (Figure 4D,H). As noise increased, there was a slight increase in the MS data support for ORFs identified from RPFs by RiboCode and RiboTaper (Figure 4D,H), due to the decreased number of total identified ORFs (Tables S2 and S3). In line with the changes in precision and F-score (Figure 4B,C,F,G), the percentage of ORFs identified by RiboTaper that were supported by MS data dropped when noise reached 60% in human cells and 40% in yeast (Figure 4D,H).

### 3.2. Identification of Translation Initiation Sites

The accurate prediction of translation initiation and termination sites is critical for the prediction of ORFs. Determining the stop codon position is relatively easier if read-through of stop codons is not permitted. In contrast, the identification of translation initiation sites (TISs) is more challenging because a stop codon can have multiple potential start codons in the same frame. Moreover, the analysis is made more complex by the inclusion of near-cognate start codons (CUG, GUG and UUG). We therefore examined and compared the accuracy of TIS prediction using a quantitative translation initiation sequencing (QTI-seq) human HEK293 dataset in which TISs were accurately determined by only sequencing the RPFs of initiated ribosomes [2]. Gao et al. identified 7974 TISs from 4195 ORFs including 3322 AUG, 1438 CUG, 572 GUG, 461 UUG and 2181 other codons, but this last subset (2181 ORFs) was excluded in the present analysis because all of the pipelines only considered NUG as the start codon. 

**Figure 5:**
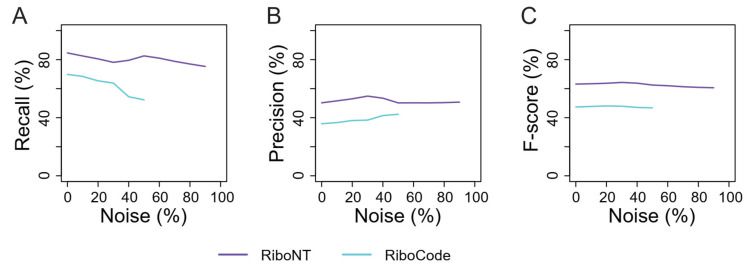
Performance of RiboNT and RiboCode in TIS identification. The recall (**A**), precision (**B**) and F-score (**C**) of TISs identified by RiboNT (purple) and RiboCode (cyan) in human using RPFs with different amounts of noise (0–90%).

We also evaluated the accuracy of the predicted TISs, benchmarked by the datasets from Gao et al. using RPFs with or without noise. For this analysis, only RiboNT and RiboCode were compared because the other two pipelines (RiboTaper and RiboWave) do not report non-AUG initiation sites. ORFs captured by QTI-seq may be from active genes with higher levels of translation, leading to a higher recall rate in this test (Figure 5A) compared to the genome-wide prediction (Figure 4A). On the other hand, because QTI-seq detected more than one TIS in many ORFs, while all the tested ORF predictors selected only the best one, the precision of these tools in this assay was considerably lower. Nevertheless, RiboNT still had higher recall, precision and F-score in this test. Without noise, 83.57% of the validated TISs were recovered by RiboNT with a precision of 51.11%, followed by RiboCode (69.85%) with a precision of 35.87% (Figure 5B; Table S4). When noise was added, the recall of RiboCode was negatively correlated to the amount of noise, whereas the precision increased as noise increased due to the decrease in the total number of predicted ORFs. As a result, the F-score of RiboCode was hardly affected by low-level noise (10–50%). 

RiboNT recall and precision were both considerably higher than those of RiboCode, and their changes were independent to the level of noise. Overall, because QTI-seq captured actively translated ORFs with abundant RPFs, both tools were robust to noise in terms of TIS identification. 

### 3.3. Identification of Small ORFs

**Figure 6:**
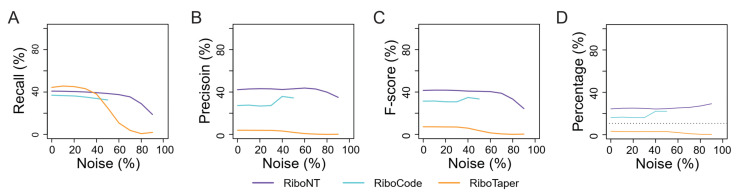
Performance of RiboNT, RiboCode and RiboTaper in sORF identification. The recall (**A**), precision (**B**), F-score (**C**) and support percentage of MS datasets (**D**) of sORFs identified by RiboNT (purple), RiboCode (cyan) and RiboTaper (orange) in the yeast genome using RPFs with varied amount of noise (0–90%). The horizontal dotted line in (**D**) indicates the percentage of MS-validated sORFs in the reference genome.

One of the most important applications of RPF-based ORF predictors is the identification of translated sORFs, which have been shown to play critical roles in translation regulation in various organisms [9,25,26]. Different tools have identified numerous sORFs in UTRs and non-coding transcripts, and many of them have been verified by MS data [12,14]. However, the rate of recall and precision of these predictions cannot be appropriately evaluated without a benchmark. In this study, we used the annotated sORFs in the yeast genome as a benchmark to evaluate the accuracy of the different tools in terms of sORF prediction. Previous studies have confirmed the translation of these sORFs in *S. cerevisiae* by fusion with green fluorescent protein (GFP) [4] or hemagglutinin (HA) tags [1], and a recent work suggested that these sORFs are functional under normal or stress conditions [27]. sORFs are very difficult to accurately identify computationally due to their short length. Compared to the high recall rates (78.05% for RiboNT; 64.98% for RiboCode; 71.34% for RiboTaper) and precision (92.43% for RiboNT; 85.28% for RiboCode; 39.27% for RiboTaper) for the prediction of annotated ORFs (Figure 4E–G), the ability of these three tools to identify sORFs was modest. Under low-noise conditions, RiboTaper had the highest sORF recall rate (44.86%) (Figure 6A; Table S5) but low precision (4.07%) (Figure 6B), resulting in a very low F-score (Figure 6C). When the noise exceeded 40%, the recall rate of RiboTaper dropped remarkably, similar to the pattern observed for the identification of annotated ORFs (Figure 4E–G). Although the RiboCode recall rates decreased, the F-scores were not affected because the reduced number of predicted ORFs resulted in increased precision (Figure 6A–C). RiboNT was tolerant to noise until it exceeded 70%. As noise increased, RPF support decreased, and as a result, the support from codon usage dominated the prediction of ORFs. In this situation of little or no support from RPFs, nonsense ORFs can arise randomly due to the short length of sORFs. We also tested riboHMM [28] and ribORF [29], but no sORFs in the yeast genome were recovered by these tools. 

Validation of the sORFs identified in each of these tests revealed that the percentage of sORFs supported by MS data was relatively small (Figure 6D) compared to the tests of annotated ORFs (Figure 4H). For example, only 10.7% of the sORFs in the reference were successfully validated (Figure 6D), while 26.6% of the annotated ORFs were validated by the MS dataset (Figure 4H). One possible explanation is that the short peptides encoded by sORFs are less likely to be included in the MS data even if they are translated at the same levels as long peptides. As the reference includes all ORFs while the RPF-based ORFs include only the translated ones, the MS support for the latter should be greater than the support for the former. The MS support for sORFs identified by RiboTaper was poor because it was smaller than that of the reference (Figure 6D) and almost 0% in this test. For RiboNT and RiboCode, the MS support increased slightly due to the decrease in sORFs under noisy conditions (Figure 6D; Table S5). 

### 3.4. Identification of Translated ORFs from Human RPFs with Poor Periodicity

**Figure 7:**
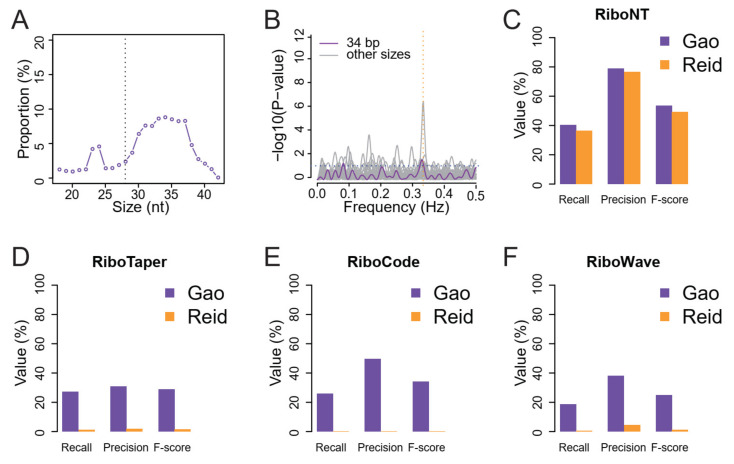
Performance of RiboNT, RiboCode, RiboTaper and RiboWave for the identification of annotated ORFs from a noisy dataset of human RPFs. (**A**) Distribution of RPFs sizes from the Reid et al. dataset with the vertical dotted line indicating the canonical 28 nt RPF size. (**B**) “Multitaper” test for periodicity of the Reid et al. RPFs (34 nt) indicates poor periodicity. The prediction of annotated ORFs from the Gao et al. (periodic) and Reid et al. (poor periodicity) datasets by (**C**) RiboNT, (**D**) RiboTaper, (**E**) RiboCode and (**F**) RiboWave.

We further compared the performance of the different tools on a dataset of human RPFs with modest periodicity that was prepared using a simplified and inexpensive method developed by Reid et al. (2015). The RPFs were notably longer than the 28 nt of canonical RPFs (Figure 7A) and had modest periodicity, as shown by the multitaper test (Figure 7B). The dataset from Reid et al. [24] contains 38.6 million RPFs and is comparable to the dataset from Gao et al. [2] with 31.9 million RPFs, which was used in the previous tests (Figure 4A–D). Using these two datasets, the outputs were compared separately for each tool (Figure 7C–F; Table S6). RiboNT had comparable performance for the two datasets (Figure 7C), while the other tools performed modestly (Figure 7D–F) for Reid et al. dataset as compared to the Gao et al. dataset.

### 3.5. Application of RiboNT to a Dataset of Arabidopsis RPFs with Poor Periodicity

**Figure 8:**
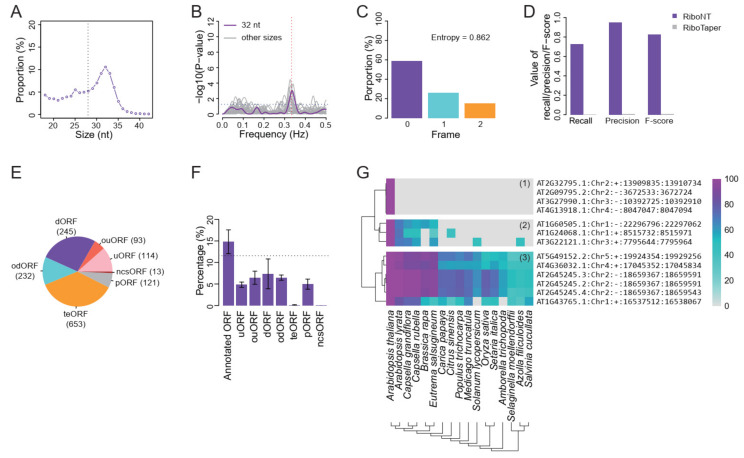
Implementation of RiboNT on a noisy RPF dataset derived from *Arabidopsis* membrane-bound polysomes. (**A**) RPF size distribution from Li et al. peaked at 32 nt with vertical dotted line indicating the canonical 28 nt RPF. (**B**) “Multitaper” test for periodicity from Li et al. (32 nt) indicates poor periodicity with high entropy (**C**). (**D**) RiboNT recovered the majority of the annotated ORFs in the *A. thaliana* genome with considerable precision, while RiboTaper did not. (**E**) RiboNT also identified several sORFs including 114 uORFs, 93 ouORFs, 245 dORFs, 232 odORFs and 13 ncsORFs. sORFs were also identified from transposable element and pseudogene transcripts. (**F**) The percentage of identified ORFs validated by the MS dataset for the varies sORF type; the error bars indicate standard deviation. The horizontal dotted line indicates the percentage of MS-validated ORFs in the reference genome. (**G**) The ncsORFs identified from this dataset can be categorized into three groups according to their sequence conservations across plant genomes. The ncsORFs in group 1 are specific to *A. thaliana* while those in group 2 are family conserved and those in group 3 are conserved from ferns to monocots and eudicots. The scale bar indicates sequence similarity.

Obtaining high-quality RPFs from membrane-bound polysomes is challenging not only in non-model organisms but also in *Arabidopsis*. Li et al. (2016) successfully extracted RPFs from *Arabidopsis* membrane-bound polysomes [10] and found that the RPF size peaked at 32 nt, instead of the canonical eukaryotic RPF size of 28 nt (Figure 8A). Additionally, the RPF periodicities were poor (Figure 8B,C). We applied RiboNT to this dataset and found that, despite the poor periodicity of the RPFs, as many as 72.64% of the annotated ORFs in the genome were successfully recovered with a precision of 92.55% (Figure 8D). In contrast, RiboTaper identified fewer annotated ORFs with a precision less than 1% (Figure 8D; Table S7). RiboCode had no output, which was in line with the tests using noisy RPFs (Figure 4A–C,E–G). A number of sORFs were also identified from this dataset, including 114 uORFs (upstream ORFs), 93 ouORFs (overlapped uORFs), 245 dORFs (downstream ORFs), 232 odORFs (overlapped dORFs) and 13 ncsORFs (ORFs on non-coding RNA) (Table S8). In addition, 653 ORFs were identified from transposable elements (teORFs), and 121 were identified from pseudogenes (pORFs) (Figure 8E). To validate these identified ORFs, we downloaded MS data of proteins extracted from *Arabidopsis* seedlings, the tissue from which these RPFs were derived. The MS dataset validated 12.86% of the identified annotated ORFs, which was higher than that of the reference (10.45%) (Figure 8F; Table S9). In line with our previous observations in yeast, only ~5% of the sORFs identified in *Arabidopsis* were also poorly supported by MS data. The support for teORFs and ncsORFs was almost 0% in this test. The former could be explained by the low levels of translation of transposable elements under the tested conditions, possibly resulting from specific but low-level translation of transposable element proteins on the endoplasmic reticulum. Only 13 ncsORFs were identified from this dataset, which were too few to be included in the MS datasets. That is, given that the average support for sORFs was ~5%, only 0.6 ncsORF-encoded proteins would be expected in the MS dataset. Nevertheless, we performed evolutionary analysis for these ncsORFs and found that their sequences were conserved among distant plant species, implying potentially important functions. The ncsORFs were divided into three groups according to the degree of sequence similarity with their homologs. Group 1 was conserved in all the analyzed genomes ranging from ferns to eudicots and monocots, group 2 was conserved only in Cruciferae, and group 3 was unique to *A. thaliana* (Figure 8G). Group 3 could represent novel ncsORFs in *A. thaliana* or false positives, but the latter seems more likely, considering the low precision (~40%) in the test of sORF identification (Figure 6B; Table S5). Despite the inclusion of potential false positives, the family-specific and overall conservation of some of these ncsORFs provided evidence supporting the accuracy of the these ORFs.

## 4. Discussion

To date, many tools have been developed to predict ORFs, particularly small ORFs, from RPFs of prokaryotes [30] and eukaryotes (see review of [7,15]). These tools allocate the translated P-sites or A-sites [31] according to the positions and offsets of RPFs, thereby determining the translated frame for a given sequence of transcripts. This strategy relies heavily on the periodicity of RPFs, and RPFs with poor periodicity would result in the false or inaccurate allocation of P-sites, leading to the false discovery of ORFs. Most, if not all, of these tools were originally developed for the study of model organisms, such as human, mouse and yeast, for which high-quality RPFs are usually achievable. However, the analysis may be complicated when Ribo-seq is applied in non-model organisms, for which high-quality RPFs may be difficult to obtain. One effective approach would be to optimize monosome isolation protocols [9], but this may not always be feasible, particularly for small research groups because Ribo-seq library construction is costly and sometimes requires special equipment [32]. To overcome these barriers, Reid et al. (2015) developed a simple Ribo-seq library construction strategy [24]. However, the RPFs obtained in their study had a non-canonical size (34 nt) and poor periodicity (Figure 7A,B), which would result in numerous unpredictable errors if the data were used to predict ORFs using the existing tools. As shown in the present analysis, the existing tools recovered very few annotated ORFs from this dataset (Figure 7D–F). While investigators are working to improve the periodicity of RPFs, the development of a noise-tolerant predictor that can make use of RPFs with poor periodicity is also important because it reduces the requirements for, and consequently extends, the application of Ribo-seq.

To this end, we developed RiboNT, a noise-tolerant ORF predictor that automatically balances and integrates the evidence from RPFs and codon usage. One of the challenges of ORF identification is the determination of TISs. Many of the existing pipelines rely heavily on the appearance of RPFs. For example, RiboTaper requires the occupancy of at least 50% of P-sites for a given ORF candidate [12]; RiboCode requires at least one RPF between the selected TIS and the next potential TIS [14]. These strategies work well when the RPFs are abundant and of high quality. However, when RPFs are sparse due to low-level ORF translation, insufficient sequencing or removal of multi-mapped RPFs, these pipelines could potentially predict shorter ORFs, particularly when near-cognate codons (CUG, GUG and UUG) are included as translation initiators. As codon usage can be assigned to each triplet within ORF candidates, the integration of the support from codon usage would alleviate the problem caused by potentially sparse RPFs. In principle, as a result of evolution, the triplets in frame 0 of a bona fide translated ORF should have greater codon usages than those in frame 1 and 2, while the triplets outside of the ORF should not. Therefore, for a given position of a stop codon, an ORF candidate starting with a false TIS, including the extended sequences beyond the true ORF, should have a *p* value greater than that of the true ORF. When the false TIS is far from the true TIS, the false ORF candidate would probably have a *p* value exceeding the cutoff (0.001 in this study) and would therefore be discarded. On the other hand, the *p* values of ORF candidates with true and false TISs could be fairly close when the TISs are close to each other, resulting in some false predictions. However, considering that translation can be initiated simultaneously from multiple sites near the annotated TIS [2,33], this kind of “false” TIS may actually be correct despite differing from the annotated TIS. This hypothesis is also supported by the increased recall in the tests of QTI-seq-validated TISs (Figure 5A) compared to that of annotated ORFs (Figure 4A).

We also compared RiboNT, RiboTaper and RiboCode, which have been widely applied in the identification of sORFs in varied species including several plants [34], for the identification of experimentally verified sORFs. For this test, Baker’s yeast (*S. cerevisiae*) provided an ideal genome dataset, in which the sORFs had been experimentally validated by labelling with detectable tags (GFP or HA) [1,4]. Our data indicate that RiboNT is more powerful in identifying both long and small ORFs than RiboTaper and RiboCode, the latter of which was previously shown to exceed many other predictors [14]. However, accurately identifying sORFs remains a challenge for RiboNT. RiboTaper showed higher recall rates in sORF identification but at the cost of precision with the detection of many false ORFs. We also tested riboHMM and ribORF for sORF identification, but both failed to identify sORFs in the yeast genome. Therefore, the use of these tools for sORF prediction may lead to high false discovery rates. It is worth mentioning that the test was conducted in a small genome, which might lead to the inflation of the performance of RiboNT. Hence, more comprehensive tests need to be further conducted when more sORFs in other genomes had been validated by experimental evidence. Our data also highlights the difficulty of sORF prediction, even with the support of RPFs. Therefore, exceptional caution should be taken in the studies of sORFs predicted without experimental supports. 

Currently available tools have been developed for the study of model organisms with diploid or haploid genome. Although RPF size is dependent on the drugs used to immobilize the polysomes [33], in most of the previous studies, the typical 28 nt RPFs in eukaryotes and RPFs close to this size (27 or 29 nt) had the best periodicity. However, a drawback of short RPFs (27–29 nt) is accurate mapping to the positions where they derive. This represents a significant problem for the study of polyploid plants, particularly important crops, such as wheat (*Triticum aestivum*, 6×), oilseed rape (*Brassica napus*, 4×), peanut (*Arachis hypogaea*, 4×) and potato (*Solanum tuberosum*, 4×). Longer RPFs could mitigate this problem, but at the same time, increased RPF size would certainly lead to decreased periodicity. There is always a trade-off between RPF periodicity and mapping accuracy. With the advantage of noise tolerance, RiboNT is able to utilize longer RPFs with minimal to no loss of power, as shown in Figure 7C. We also applied RiboNT to a noisy dataset of RPFs isolated from membrane-bound polysomes. It was successful in recovering most of the annotated ORFs with considerable precision from this noisy dataset suggests that RiboNT is highly tolerant to noise. In addition to annotated ORFs, RiboNT also identified novel ncsORFs that was evolutionary conserved among diverse plant species, ranging from ferns to monocot and eudicot plants.

Considering its high noise tolerance, RiboNT should facilitate the utilization of low-quality and longer RPFs for ORF identification, which will greatly expand the application of Ribo-seq.

## Data Availability

RiboNT is available in the GitHub repository (https://github.com/songbo446/RiboNT/, accessed on 13 July 2021).

## Supplementary Materials

The following are available online at https://www.mdpi.com/article/10.3390/life11070701/s1, Table S1: The weights of RPFs and codon usage in the prediction using noisy RPFs, Table S2: Comparison of pipelines in identification of annotated ORFs in human, Table S3: Comparison of pipelines in identification of annotated ORFs in *S. cerevisiae*, Table S4: Comparison of pipelines in identification of TISs, Table S5: Comparison of pipelines in identification of annotated sORFs in yeast, Table S6: Identification of annotated ORFs in human genome using low-quality RPFs, Table S7: Identification of annotated ORFs in A. thaliana genome using low-quality RPFs, Table S8: small ORFs predicted from noisy RPFs of Arabidopsis, Table S9: ORFs identified in A. thaliana from low-quality RPFs.
